# Nudging to prevent the purchase of incompatible digital products online: An experimental study

**DOI:** 10.1371/journal.pone.0173333

**Published:** 2017-03-10

**Authors:** Gabriele Esposito, Penélope Hernández, René van Bavel, José Vila

**Affiliations:** 1 Joint Research Centre, European Commission, Seville, Spain; 2 Center for Research in Social and Economic Behavior (ERI-CES), University of Valencia, Valencia, Spain; Middlesex University, UNITED KINGDOM

## Abstract

Ensuring safe and satisfactory online shopping activity, especially among vulnerable consumers such as elderly and less educated citizens, is part of a larger set of consumer policy objectives seeking to strengthen trust in the electronic marketplace. This article contributes to that goal by testing the effectiveness of nudges intended to prevent the purchase of 'incompatible' digital products (i.e., those which cannot be used with the devices owned by consumers or the systems they operate). We ran a computerised lab experiment (n = 626) examining three types of nudges, the effects of age and education, and interaction effects between these variables and the nudges. Results show that emotive warning messages and placing incompatibility information at the checkout page rather than earlier in the purchasing process were effective in reducing the purchase of incompatible goods. Age was also a relevant factor: older participants were more likely to purchase incompatible goods. In addition, there was an interaction effect between all nudges and age: two nudges exacerbated the effect of age, while another mitigated it. These results suggest nudges can be an effective policy tool, confirm a generational gap in online behaviour, and highlight how nudges can moderate the effect of socio-demographic variables.

## Introduction

Online shopping has long entered a consolidation phase [1]. No longer just for early adopters, users are more diverse, and include sectors of the population who might until recently not have been familiar with conducting transactions over the Internet. Consumer policy has had to react accordingly, ensuring that the rules and regulations which apply to online shopping are up to date and effective in protecting all consumers–especially the most vulnerable—from abuse and from making mistakes which might undermine their trust in eCommerce as a whole.

In the EU, a series of policy initiatives, most notably the Consumer Rights Directive (2011/83/EU), pay particular attention to online shopping. These initiatives dovetail with another prominent EU policy priority, namely reinforcing the Digital Single Market, for which a trusted and secure online shopping environment is required [2].

The Consumer Rights Directive includes a general requirement that consumers be informed about the main characteristics of the products they are purchasing and various other aspects of electronic transactions. Such information is often missing, complex, unclear or hidden, despite existing relevant legislation [3]. Specifically, with regard to digital goods (such as films or games, which can be downloaded instantly), it requires traders to provide consumers with pre-contractual information on the functionality and interoperability of products. Functionality refers to the way the digital content can be used, including technical restrictions; interoperability to the standard hardware and software with which the digital content is compatible.

However, simply requiring traders to provide information might not be enough. Consumers must be able to find and interpret this information and use it to make good decisions. Without this ability, consumers will make mistakes, leading to disappointment, frustration, and ultimately lack of trust in online shopping. The evidence from behavioural economics suggests that the way information is presented can either facilitate or hamper decisions that are in the consumer's own best interest. This study was based on this assumption and sought to test how small changes to a website's design can affect purchase behaviour, with particular attention to the interaction with socio-demographic characteristics of the participant. In addition to contributing to the scientific literature, this article contributes to the evidence base from which policy-makers can draw upon to strengthen trust in the electronic marketplace.

## Conceptual framework

This study applies behavioural insights to policy-making, in the wake of a growing trend [4, 5, 6, 7, 8, 9]. It aimed to contribute to guidelines, elaborated by the European Commission, to EU member states on how to implement certain provisions of the Consumer Rights Directive (2011/83/EU). The applied nature of the study meant it welcomed behavioural insights from different schools of thought in the behavioural sciences, rather than adhering to a single theoretical framework.

The behavioural economics approach to policy issues is one called *libertarian paternalism*, whereby policy-makers make small, appropriate changes in the environment where a choice takes place (the *choice architecture*) to gently persuade (or *nudge*) people's behaviour [10]. It is libertarian in allowing consumers the freedom to do as they please, but paternalistic in facilitating the choice which is presumed to be in the consumer's best interest. In an on-line environment, the choice architecture includes such features as website design, warnings, and defaults [11].

In the case of digital goods, behavioural insights can help identify the elements to be included in the choice architecture of websites which sell them in order to nudge consumers into making satisfactory digital product purchases [10]. The premise is to change automatic behaviour, which characterises the repetitive activities of daily digital activities, such as email or Internet browsing. This routinized behaviour is guided by *fast thinking*, and can be changed without necessarily evoking a change in conscientious behaviour, governed by *slow thinking* [12].

Two behavioural insights were translated into nudges aiming to help consumers make compatible purchases: (a) highlighting the emotions associated with a particular behaviour leads to more effective warning messages [13, 14] and (b) visual images are more effective than text in leading to behaviour change [15, 16]. The experiment also tested whether providing relevant information at an early stage in the purchase process led to different behaviour than placing it at a final (i.e. checkout) stage. This test was specifically requested as an input to the policy-making process.

An analysis of the effect of socio-demographic factors and how they might interact with nudges was another objective of the study. With online shopping becoming a mainstream activity, the population of online shoppers has grown and incorporated a great variety of people. Against this setting, consumer policy has to consider that initiatives intended to protect consumers and increase their trust in the online shopping environment might have to display some variety as well. It is not possible, *a priori*, to know whether everyone will react similarly to a policy intervention, or whether these reactions will vary according to socio-demographic segment. This study, therefore, sought to answer the following research questions: (a) will the nudges work? (b) will age and education have an effect on behaviour? and (c) will the effects of age and education be affected, in turn, by the nudges? In so doing, the study aimed to contribute to future policy interventions aimed at protecting the consumer and generating trust in the online environment.

## Nudges, socio-demographics and hypothesis development

### Nudges

The nudges seeking to limit the number of incompatible purchases had to be realistic (i.e. their implementation had to be feasible in a real world environment, beyond the laboratory), policy-relevant (i.e. they had to represent an option that was being discussed in the EU consumer policy agenda) and theory-driven (i.e. there had to be justification for a nudge of this kind in the literature). In addition, only a few nudges could be tested, because of budget constraints.

Three nudges were selected for this study: a *warning message*, the *style* in which product information was provided (text vs. logos), and a change in the stage in the purchasing process at which this information was provided (*information placement*).

Warning messages have been shown to be an effective nudge, as they attract the consumer’s attention, which can be overloaded by stimuli in the online environment [11], [17]. In this study, a warning message was placed on the checkout page, reminding consumers to select compatible purchases. It was intended to heighten perceived purchase risk, i.e. people's assessment of the probability of making a mistake and the severity of its consequences [18, 19], which should influence their behaviour [20]. Specifically, increased perceived purchase risk should lead consumers to search for more product information [21].

Two types of warnings were tested: a *traditional message* and a message appealing to the emotions of the user (*emotive message*). The introduction of emotion followed the general expectation that both *anticipated* and *anticipatory* emotions impact on consumers’ cognitive processes [22]. Anticipated emotions refer to the expectation of emotions that will be experienced in the future, as a result of a decision. People therefore work these expectations into their decision-making process [23, 24, 25]. Anticipatory emotions, on the other hand, are immediate visceral reactions in the face of a risk or uncertainty [26]. We sought to elicit both types of emotion in the emotive warning message, expecting that it would lead to less impulsive and more cautious purchasing [27].

The second nudge was to use logos instead of text for providing key information on compatibility. A logo is an essential part of a company's image, and is intended to attract consumer attention and stimulate instant company recognition [28]. In the online domain, where consumer attention is highly sought after, logos have become more important than ever before [29]. Conveying compatibility information visually through logos should therefore be more effective in getting people's attention and making them choose compatible purchases.

A third nudge consisted of placing product compatibility information on the checkout page instead of the page where detailed information about the product is usually provided (the 'product description page'). The reason for placing compatibility information at the checkout page was that this is the final, irrevocable, step in the shopping process, which should make buyers more attentive to key information.

### Socio-demographics

There is ample evidence that highlights the role of socio-demographic factors in accounting for online shopping behaviour [30, 31, 32, 33]. On the other hand, some research claims that, once individuals become experienced e-shoppers, they behave in similar fashion regardless of their socio-demographic characteristics [34]. There is sufficient indication, at any rate, that some socio-demographic factors might be relevant and need to be considered in an analysis of online shopping. In our study, we focused on age, education and gender.

#### Age

When discussing shopping behaviour generally, we can expect older consumers, because of their greater shopping experience, to be capable consumers, displaying a smarter and more sophisticated choice process [35, 36, 37]. Younger consumers, by contrast, may be more likely to use simple rules of thumb [38]. With regard to the propensity to shop online, the findings in the literature have been inconsistent. Some research shows that elder people are more likely to buy online than younger individuals, but others have found the opposite [39, 1].

With regard to making incompatible online purchases, however, it would seem that older consumers are at a disadvantage. The literature suggests that increased age is associated with difficulties in processing information and using the Internet effectively. This is partly because they are less likely to be Internet users in the first place. However, they also tend to lack the appropriate social networks to help them understand and navigate the electronic marketplace [40]. This is particularly true of over 65 year-old's, who grew up and lived their adult lives in very different circumstances and who now find themselves living in a world that has been changed by digital technology and which effectively has invalidated their 'experience of a lifetime' [41, 42]. Generally speaking, older people are less interested in new technologies and are more likely to be overwhelmed by them [31, 33]. In this study, we expected older participants would be more prone to making incompatible product purchases.

#### Education

With regard to education, the literature suggests that higher educated people handle the potential complexities that might arise when navigating online better. They display lower levels of computer anxiety [43] and show a greater ability to learn when handling a sophisticated system [44].

Regarding online shopping, previous studies show that more educated people are more likely to shop online than less educated people [45]. They were also more likely to be satisfied with the online shopping experience, suggesting that they were able to do it successfully [46]. Regarding this study, therefore, their greater competence in online shopping should make them less likely to make incompatible purchases.

#### Gender

There are some notable differences between men and women when it comes to online shopping. For one, they prefer different products. Female consumers are more likely to buy clothes, jewellery and accessories than male consumers [47], who are more likely to buy electronics, sporting goods and car equipment [1]. But they also differ in their sopping orientation. Women have displayed higher shopping enjoyment, brand and fashion consciousness, and shopping confidence than men, who in turn have showed higher convenience and time consciousness than women. This suggests that women might be more hedonic and men more functional in their online shopping behaviour [47].

Women and men also differ in their attitudes to online shopping. One survey suggests it may be more appealing to men, as they showed significantly higher cognitive, affective and behavioural attitudes to online shopping compared to women [48]. Also, women seem to perceive a higher level of risk in online purchasing than men. Interestingly, having a site recommended by a friend reduces this risk perception and increases willingness to buy–and this effect is stronger among women than men [49]. All of these insights led us to include gender as a potentially relevant variable, but in an exploratory fashion (i.e. they did not translate into a working hypothesis).

### Hypothesis development

In sum, the study was guided by the following hypotheses regarding the effectiveness of nudges in preventing incompatible purchases and the possible effect of socio-demographic factors:

- H_1_: the traditional warning message will lead to fewer incompatible purchases than the control condition (no warning at all).
- H_2_: the emotive warning message will lead to fewer incompatible purchases than the control condition (no warning at all).
- H_3_: the emotive warning message will lead to fewer incompatible purchases compared to the traditional warning message.
- H_4_: information about product compatibility provided through logos will lead to fewer incompatible purchases than key information provided through text.
- H_5_: information about product compatibility presented on the checkout page will lead to fewer incompatible purchases than information presented on the product description page.
- H_6_: age will have a positive impact in the purchase of incompatible products: older participants will purchase a higher proportion of incompatible goods.
- H_7_: education will have a negative impact in the purchase of incompatible products: more educated participants will purchase a lower proportion of incompatible goods.

The study also tested for interaction effects between the socio-demographic variables (age and education) and the experimental treatments (warning message, style and information placement). In other words, if there were a effect of age and education on the purchase of incompatible products, would this effect be the same in all experimental conditions? Or could it be that this effect is increased (or reduced) in the presence of a given nudge? Since it is difficult to predict *ex ante* the direction and magnitude of these interaction effects, this part of the investigation was exploratory and not guided by specific hypotheses.

## Methodology

### Participants

We conducted a computer laboratory experiment with 626 participants at the Laboratory of Research on Behavioral and Experimental Economics (LINEEX) in Valencia, Spain. Ethical approval was granted by the Human Research Ethical Committee of the Experimental Research Ethics Commission, University of Valencia. Instructions, mock websites and questions were in Spanish. The sample was representative of Spaniards who had purchased a digital good online at least once, according to the information provided by INE (*Instituto Nacional de Estadística de España*, the Spanish National Statistics Institute) in its Survey on Equipment and Use of ICT in Households, 2012.

The sample was obtained from a subject pool managed by LINEEX of over 25,000 volunteers. The recruitment system at LINEEX opened a call on its web page, only visible to those participants already registered in the database. Participants had to be knowledgeable about online shopping, and answered several questions to confirm their knowledge in the registration process. They also had to be new to LINEEX experiments (i.e. they could not have been a participant in a previous experiment at the same laboratory). Participants could register to one, and only one, session through the web page. They were randomly assigned to experimental treatments until the representative quotas for age and gender were completed in each treatment. After that, no more participants of the age group or gender whose quota had been reached were allowed to register. Two categorical age groups were used: 18–34 and ≥35. This resulted in a sample of 243 men aged 18 to 34, 147 men aged 35 and over, 77 women aged 18 to 34 and 159 women 35 and over. The oldest participant was 76 years old.

### Experimental design

**Fig 1:**
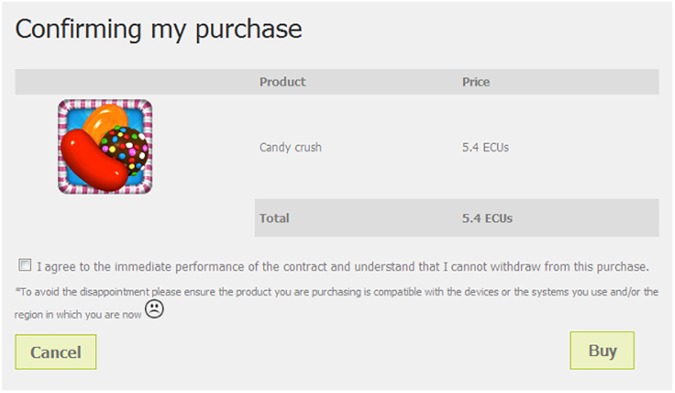
Emotive warning message on the checkout page. Reprinted from https://www.flickr.com/photos/146051417@N08/ under a CC BY license, with permission from LINEEX—Universitat de Valencia, original copyright 2016. Additional screenshots of webpages used in the experiment are available in S1 Screenshots.

**Fig 2:**
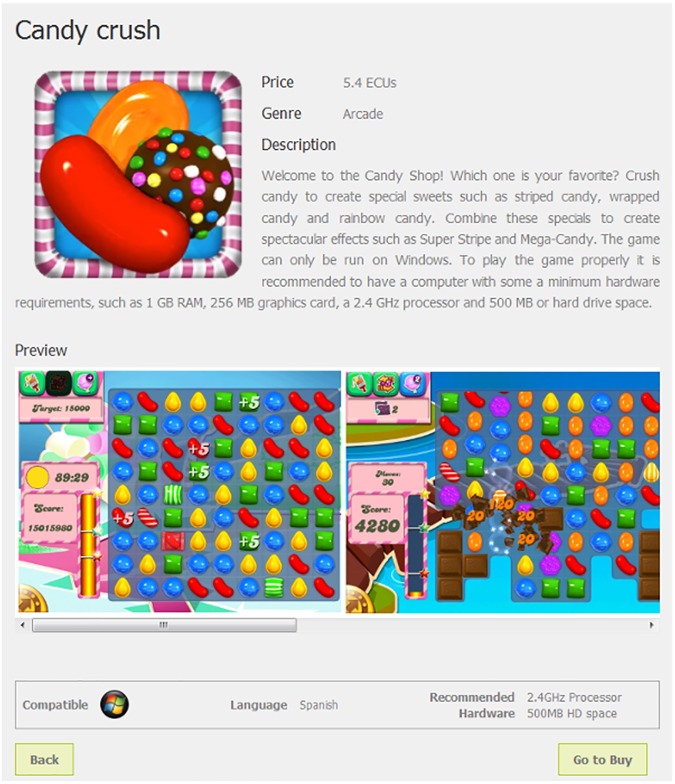
Compatibility information provided as a logo on the product description page. Reprinted from https://www.flickr.com/photos/146051417@N08/ under a CC BY license, with permission from LINEEX—Universitat de Valencia, original copyright 2016.

Participants were randomly assigned to one and only one of the 12 experimental conditions. This process controlled for gender and age in order to maintain the representativeness of each treatment group. Once a given quota was fulfilled in a session, the recruitment system at LINEEX did not allow for additional registrations fitting that profile. The result was a 3 x 2 x 2 matrix which combined the following elements (see illustrative screenshots in Figs 1 and 2):

- Warning messages: No warning message, *traditional* warning message and *emotive* warning message.
- Style: Compatibility information provided as text or as a logo (Windows or Apple for games; a US or EU flag for films).
- Information placement: Compatibility information presented at different stages of the purchasing process (on the product description page or on the checkout page).

These experimental treatments were designed paying attention to what could be feasibly achieved in an online shopping environment. Messages had to be short and sweet, and could not burden the shopping process nor cognitively overload the consumer. The anticipated emotion of disappointment due to an incompatible purchase was heightened with a brief subordinate clause ('To avoid disappointment…'). The anticipatory emotion of concern was conveyed with an emotion icon (emoticon) of a sad-looking face. Emoticons are often used as nonverbal cues to reinforce the emotive content of computer-mediated messages [50].

The traditional warning read: 'Please ensure that the product you are buying is compatible with the devices and systems you use'. The emotive warning message read: 'To avoid disappointment, please ensure that the product you are buying is compatible with the devices and systems you use and the region in which you are'. This additional mention of the region was an oversight, and limited the validity of a comparison with the traditional message (since, strictly speaking, the impact of the emotive component was not fully isolated), though not the analysis of the moderating effect of socio-demographic variables. To isolate the effect of the expressions conveyed by the emoticon, the traditional message contained an empty circle of the same size as the sad emoticon.

### Implementation of the experiment

The experiment took place over 13 sessions in October 2013. Participants started the experiment by viewing a large Microsoft Windows logo on their computer screens. A member of staff, who was the same for all 13 sessions, read the instructions out loud (participants could also read them at the same time) and asked participants if they could see the Windows logo on their desktops. This step was vital in the experiment, as it let participants know, albeit not explicitly, that they were using a computer with a Windows operating system.

Participants were told they had some virtual currency to purchase games and films on an e-commerce website over a period of 10 minutes, and that plenty of information was available to help them choose the best options. They were also informed they would have 15 minutes to enjoy their purchased products at the end of the shopping process. Moreover, at the end of the experiment, the amount of virtual currency spent on products that were effectively used (i.e. which were compatible) would be converted into real money. It was made very clear to the participants that, in order to be paid, they had to *use* the products, meaning they had to play a game or watch a movie for a minimum amount of time. At the end of the process, participants were paid according to their performance and escorted off the laboratory premises.

In all 12 experimental conditions, the information available to participants was clearly written on the description page for each digital product. This information included the products' main characteristics and, most importantly, information on their functionality and interoperability (such as the compatible operating system, the country in which the product could be used, and technical details such as RAM, minimum processor speeds or graphic requirements). Each product had a prominently placed box, which summarised its compatibility with the operating system for games (Microsoft Windows or Apple) or with the geographical region for movies (Europe or USA). This information differed according to treatment groups described earlier: in some, compatibility information was only included on the checkout page, not on the product description page. In others, this information was presented in one of two formats: as text, or with logos. The product description pages also included some screenshots of the product, reviews and user comments (which participants were not able to amend). Effort was made to ensure that user comments for all products were qualitatively comparable, in order to avoid any effect due to word-of-mouth [51, 52].

Participants had enough virtual currency to purchase five products, which could be freely chosen from a total of 10 films and 10 games. Participants in a particular age group paid the same price for all products. However, in order to facilitate recruitment of the over 35 year olds, these participants were paid higher prices and were given more virtual money than participants under 35. In particular, the under 35 year olds were given 18 units and paid 3.6 units per product (leading to them to receive a maximum payment of €18) whereas the over 35's were given 27 units and paid 5.4 units per product (leading them to receive a maximum payment of €27). No fixed amount of money was guaranteed to participants. However, a minimum payment of €10 was established as a courtesy to those participants who would have been paid less on the basis of their performance.

Five films and five games were not compatible with the system in use, i.e. five films could only be watched in the US and five games could only be played on an Apple computer (hence the importance of the large Microsoft Windows logo on computer desktops at the start of the experiment). For the compatibility of films, this was not necessary as people obviously knew they were in Spain. The order of products on the homepage was identical for each participant, starting with an incompatible product in the top left corner for both movies and games.

The choice of films, also chosen in [53], and games as products rather than music was due to the greater role played by information in their purchase (music files are rarely incompatible on a PC). They were also preferred to e-books as they could be used more immediately. Using two categories of digital products, moreover, allowed a wider fit with the tastes and demographic characteristics of participants.

After confirming a purchase, participants could not cancel it. As they were only allowed to use the products at the end of the shopping simulation, they did not know whether they had made incompatible purchases until they reached this phase.

When they had spent all their virtual currency, or after 10 minutes from the start, participants received confirmation of how many of the products they had purchased were compatible and were paid accordingly. In this way, the experiment was fully incentivised. Participants had the incentive to buy goods they liked (as they knew they would have to actually experience them), and something that actually worked on the lab computer (otherwise they would not get paid for it).

## Results

The purpose of the experiment was to test if differences in the choice architecture of an online shopping website could lead to more compatible online product purchases and how this effect interacted with the profile of the purchaser, as formalised in Hypotheses 1 to 7. The empirical evidence to test these hypotheses is provided by the specification and estimation of an ANCOVA (ANalyisis of the COVAriance) model, which relates the behavioural measure, i.e. the number of incompatible goods purchased, with the different information frames presented in the treatments and the socio-demographic profile of the buyer. ANCOVA is required due to the presence of both continuous (age) and categorical (all the other) variables. To analyse potential interaction effects in the purchase of incompatible products, the model also included interaction terms between treatments and between treatments and socio-demographic variables.

The model's dependent variable is the number of incompatible products purchased, out of a maximum of five. The independent variables are determined by (1) the variable treatments: warning message (three levels), style (two levels), information placement (two levels) and (2) the profile characteristics of the purchaser: age (continuous), gender (two levels), education (four levels), employment (five levels) and frequency of online purchase of digital products in the last 12 months (five levels). Age was introduced in the model as a covariate since it is a continuous variable, and the rest of the categorical variables were introduced as fixed factors. Table 1 describes the variable treatments and their levels.

**Table 1:** Variable Treatments and Levels.

Variable	Treatments	Description
Warning message	No warning (control)	-
	Traditional warning	"Please ensure that the product you are buying is compatible with the devices and systems you use. ○"
	Emotive warning	"To avoid disappointment, please ensure that the product you are buying is compatible with the devices and systems you use and the region in which you are. ☹"
Information placement	Product description page	Compatibility information is presented at the product description page
	Checkout page	Compatibility information is presented at the checkout page
Style	Text	Text says "Windows", "Apple", "Europe", or "United States"
	Logos	A small icon showing the Microsoft Windows logo, the Apple logo, the European flag, or the flag of the United States

The ANCOVA F-test is a useful tool to test the research hypotheses. This test can be applied without the normality assumption for large samples, like the one in this experiment [54, 55]. The use of the test requires, however, the independence of the observations and homogeneity of variance (homoscedasticity). The between-subjects design of the experiment, where each subject participated in only one treatment, ensures independence between observations. Finally, the Levene Test was applied to test the homoscedasticity hypothesis. The *p*-value for the Levene Test was 0.804 and the null hypothesis was not rejected. Therefore, the statistical assumptions required for the application of ANCOVA F test and use of ANCOVA model were satisfied and this methodology could be properly used to analyse the data.

The global ANCOVA F-test guaranteed the existence of significant effects in the model (p-value = 0.002). The detailed results of the ANCOVA for the treatments and socio-demographic variables are presented in Table 2. Regarding the treatments, the table shows that the effect of *information placement* is significant (p-value = 0.011) and the effect *style* is not (p-value = 0.226). However, the F-test provided no conclusive evidence on the effect of the three-level *warning message* treatment (p-value = 0.076). *Age* is the only socio-demographic variable that is significantly related with the dependent variable (p-value = 0.008).

**Table 2:** ANCOVA for the Treatments and Socio-demographic Variables.

Factor	Source	Partial SS	df	F	*p*-value
Treatment variables (categorical)	Style	2.226	1	1.469	0.226
	Warning message	7.829	2	2.583	0.076*
	Information placement	9.936	1	6.556	0.011**
Profile variable (categorical)	Gender	2.807	1	1.852	0.174
	Education	4.058	3	0.893	0.445
	Employment	4.985	4	0.822	0.511
	Digital products	6.142	4	1.013	0.400
Profile variable (continuous)	Age	10.869	1	7.171	0.008***

Level of significance.

*** *p* < 0.01.

** *p* < 0.05.

* *p* < 0.10; n = 626.

The estimated values of the parameters of the ANCOVA model are required to test the research hypotheses and complete the analysis of the effect of *style*. For the sake of interpretability, and due to the large number of parameters of the model, Table 3 only presents those parameters significant at the 0.05 level. In particular, the analysis yields the following results:

- The coefficient associated to the traditional warning message is not significantly different to zero (*p-value* = 0.482), meaning H_1_ is not supported. There is no evidence that including a traditional warning message is more effective than including no warning at all.
- The emotive warning message is more effective than no warning message (p-value = 0.015). H_2_ is supported. Specifically, the inclusion of an emotive warning message reduces the purchase of incompatible products by 0.908 units. Moreover, since the emotive message was effective and the traditional not, H_3_ is supported as well.
- The *style* in which the compatibility information is presented (text or logos) has no influence on the number of incompatible products purchased by the subjects (p-value of the F test = 0.226, see Table 2). H_4_ is not supported.
- Compatibility information at the checkout page leads to fewer incompatible purchases than the same information placed at the product description page. H_5_ is also supported (p-value = 0.049).
- Age exhibits a positive (estimated coefficient = 0.026) and significant (p-value = 0.004) relation with the purchase of incompatible products. The older the participant, the greater the number of incompatible digital goods purchased. H_6_ is supported.
- Education has no significant effect on behaviour. H_7_ is not supported.

**Table 3:** ANCOVA at the 0.05 Level.

Parameters	B	Sd. Error	t	Sig
Intersection	1.416	0.504	2.810	0.005**
[Information placement = product description page]	0.642	0.325	1.975	0.049**
[Information placement = checkout page]	0ª			
[Warning message = emotive]	-0.908	0.374	-2.428	0.015**
[Warning message = traditional]	-0.270	0.383	-0.704	0.482
[Warning message = no warning]	0ª			
Age	0.026	0.009	2.895	0.004***
[Information placement = product description page] x age	-0.016	0.007	-2-217	0.027**
[Information placement = checkout page] x age	0ª			
[Style = logos] x age	-0.015	0.007	-2.109	0.035**
[Style = text] x age	0ª			
[Warning message = emotive] x age	0.021	0.009	2.367	0.018**
[Warning message = traditional] x age	-0.006	0.009	-0.665	0.506
[Warning message = no message] x age	0ª			

Level of significance.

*** *p* < 0.01.

** *p* < 0.05.

^a^ Parameter has been assigned a value of zero because it is redundant.

The analysis also uncovered significant interaction effects between experimental treatments and age (but not education). The direct effect of age on incompatible purchases is increased by the inclusion of an emotive warning message (p-value = 0.018; estimated coefficient = 0.021) and by presenting compatibility information at the checkout page (p-value = 0,027; estimated coefficient = 0.016). The opposite was true of using logos instead of text style: in this case, the nudge did help mitigate the effect of age (p-value = 0.035; estimated coefficient = -0.015).

## Discussion and implications

This has tested how small changes in a website's design can induce more effective on-line purchases of digital goods. Data was obtained from a computerised laboratory experiment (n = 626) considering 12 treatments and paying special attention to the possible interactions of these treatments with age and education. The 12 experimental conditions were the result of a 3 x 2 x 2 matrix which combined warning messages (no warning message, *traditional* warning message and *emotive* warning message); style (compatibility information provided as text or as logo); and information placement (compatibility information presented at different stages of the purchasing process).

Results show that two nudges reduced the purchase of incompatible goods: an emotive warning message (as opposed to no warning message) and placing compatibility information on the checkout page (rather than on the product description page). A traditional warning message was no more effective than no message at all, and the style in which compatibility information was presented also did not have an effect. Age had a direct effect (older participants make more incompatible purchases), but not education.

The result of the emotive warning message is encouraging, and confirms that subtly tapping into people's emotions increase the effectiveness of nudges. The impact of placing compatibility information at the checkout stage confirms perhaps what we believe intuitively, namely that people will pay more attention in the final, irrevocable step, even though there is little in the literature to support this claim. Finally, the impact of age was as expected: older participants are less familiar with the Internet (they were not born into a fully-digital society) and face more difficulties in navigating it appropriately.

The interaction of this age effect with all nudges, regardless of their strength, was particularly insightful. It was stronger when combined with emotional warning messages and compatibility information placed at the checkout page, but weaker when logos were used instead of text. These results suggest a generational gap in response to nudges: while some nudges might be effective in reducing incompatible purchases in general, they may not be effective in mitigating the deleterious direct effect of age.

What could account for the inability of the emotive warning message and the placement of compatibility information at the checkout to mitigate the effect of age, given that they had proven to be effective in preventing incompatible purchases generally? Perhaps the use of emoticons (now superseded by *emojis*) in the emotive message appealed more to younger, who are more accustomed to them. Older people, on the other hand, might not interpret these signs appropriately. Regarding information at the checkout page, maybe older people are more patient and study information better at earlier stages of the purchasing process compared to younger people. Reassuringly, logos work well as nudges in general and to mitigate the effect of age as well.

These results have policy implications for the design of web interfaces and regulation that seeks to protect vulnerable consumers. Specifically, age is an important factor which mediates the effect of changes in user interfaces on behaviour. Therefore, regulation that seeks to protect vulnerable consumers should take note. Policy-makers should seek to test consumer protection initiatives specifically on the intended age group, as findings from other groups might not be generalizable.

In sum, nudging can be an effective policy tool for encouraging safe and satisfactory online shopping. This article offers just a glimpse of what is achievable. More testing on how nudges affect online behaviour is needed to generate a body of knowledge and guidelines on the appropriate design of online shopping sites.

## Supporting information

S1 Dataset Full Data Set of the Experiment. (ZIP)

S1 Screenshots Additional Screenshots of Webpages used in the Experiment. (PDF)

## References

[1] Burkolter D, Kluge A. Online consumer behavior and its relationship with socio-demographics, shopping orientations, need for emotion, and fashion leadership. J of Bus and Media Psychol. 2011; 2(2): 20–28.

[2] European Commission. A digital single market strategy for Europe: analysis and evidence. SWD(2015) 100 final. Available online at http://eur-lex.europa.eu/legal-content/EN/TXT/HTML/?uri=CELEX:52015SC0100&from=EN.

[3] Europe Economics. Digital content services for consumers: assessment of problems experienced by consumers (Lot 1), Report 4: Final report. London: Europe Economics. Available online at http://ec.europa.eu/justice/consumer-marketing/files/empirical_report_final_-_2011-06-15.pdf.

[4] Obama B. Executive order–using behavioural science insights to better serve the American people (Executive Order 13707) Washington, DC: The White House; 2015.

[5] Lunn P. Regulatory policy and behavioural economics OECD Publishing; 2014

[6] Lourenço JS, Ciriolo E, Almeida SR, Troussard X. Behavioural insights applied to policy: European report 2016. EUR 27726 EN; 2016.

[7] van Bavel R, Herrmann B, Esposito G, Proestakis A. Applying behavioural sciences to EU policy-making, JRC Scientific and Policy Reports EUR 26033 EN; 2013. Available at http://ftp.jrc.es/EURdoc/JRC83284.pdf.

[8] World Bank. World development report 2015: mind, society, and behaviour; 2015. Available online at http://www.worldbank.org/en/publication/wdr2015.

[9] Kraft-Todd G, Yoeli E, Bhanot S, Rand D. Promoting cooperation in the field. Curr Opin in Behav Sci. 2015 6 30; 3:96–101.

[10] Thaler R, Sunstein C. Nudge: improving decisions about health, wealth, and happiness London: Penguin; 2008

[11] Sunstein CR. Nudging: a very short guide. J of Consumer Policy, 2014;37(4): 583–588.

[12] Kahneman D. Thinking, fast and slow London: Penguin; 2011.

[13] Cameron AM. Social media and organ donation: the Facebook effect. J of Leg Medicine. 2015; 36(1): 39–44. 10.1080/01947648.2015.1049916 26208612

[14] Aichholzer G. Citizen participation in climate governance In: Aichholzer G, Kubicek H, Torres L, editors. Evaluating e-Participation. Springer International Publishing; 2016 pp. 47–62.

[15] Choe EK, Jung J, Lee B, Fisher, K. Nudging people away from privacy-invasive mobile apps through visual framing. In IFIP Conference on Human-Computer Interaction. Berlin, Heidelberg: Springer; 2013. pp. 74–91.

[16] Newell RG, Siikamäki J. Nudging energy efficiency behavior: the role of information labels. J of the Assoc of Environ and Resour Economists. 2014; 1(4): 555–598.

[17] Thompson DV, Hamilton RW, Rust RT. Feature fatigue: when product capabilities become too much of a good thing. J of Mark Res. 2005; 42(4): 431–442.

[18] Cunningham M. The major dimensions of perceived risk In: Cox D, editor. Risk taking and information handling in consumer behavior. Boston: Harv Univ Press; 1967 pp. 82–111.

[19] Jacoby J, Kaplan LB. The components of perceived risk. Adv in Consum Res. 1972; 3(3): 382–383.

[20] Sheeran P, Harris PR, Epton T. Does heightening risk appraisals change people’s intentions and behavior? A meta-analysis of experimental studies. Psychol Bul. 2014; 140(2): 511–543. 10.1037/a0033065 23731175

[21] Spiekermann S, Paraschiv C. Motivating human–agent interaction: transferring insights from behavioral marketing to interface design. Electron Commerc Res. 2002; 2(3): 255–285.

[22] Bagozzi RP, Gopinath M, Nyer PU. The role of emotions in marketing. J of the Acad of Mark Sci. 1999; 27(2): 184–206.

[23] Bell DE. Disappointment in decision making under uncertainty. Operations Res. 1985; 33(1): 1–27.

[24] Loomes G, Sugden R. Disappointment and dynamic consistency in choice under uncertainty. The Rev of Econ Stud. 1986; 53(2): 271–282.

[25] Perugini M, Bagozzi RP. The role of desires and anticipated emotions in goal‐directed behaviours: broadening and deepening the theory of planned behaviour. Br J of Soc Psychol. 2001; 40(1): 79–98. 11329835 10.1348/014466601164704

[26] Loewenstein GF, Weber EU, Hsee CK, Welch N. Risk as feelings. Psychol Bull. 2001; 127(2): 267 11316014 10.1037/0033-2909.127.2.267

[27] Hill RP, Gardner MP. The buying process: effects of and on consumer mood states. Adv in Consum Res. 1987; 14(1): 408–410.

[28] Vartorella W. Doing the bright thing with your company logo. Advert Age. 1990; 61(2): 31.

[29] Jansen BJ, Zhang L, Mattila AS. User reactions to search engines logos: Investigating brand knowledge of web search engines. Electron Commer Res. 2012; 12(4): 429–454.

[30] Burke RR. Technology and the customer interface: what consumers want in the physical and the virtual store. J of the Academ of Mark Sci. 2002; 30: 411–432.

[31] Monsuwé TPY, Dellaert BGC, de Ruyter K. What drives consumers to shop online? a literature review. Int J of Serv Ind Manag. 2004; 15: 102–121.

[32] Suki NM. Gender, age, and education: do they really moderate online music acceptance? Commun of the IBIMA; 2011.

[33] Venkatesh V, Morris MG. Why don’t men ever stop to ask for directions? Gender, social influence, and their role in technology acceptance and usage behaviour. MIS Qin. 2000; 24(1): 115–140.

[34] Hernández B, Jiménez J, Martín MJ. Age, gender and income: do they really moderate online shopping behaviour? Online Inf Rev. 2011; 35(1): 113–133.

[35] Berg L. Consumer vulnerability: are older people more vulnerable as consumers than others? Int J of Consum Studies. 2015; 39(4): 284–293.

[36] Sherman E, Schiffman LG, Mathur A. The influence of gender on the new‐age elderly's consumption orientation. Psychol Mark. 2001; 18(10): 1073–1089.

[37] Shukla P, Banjerjee M, Adidam PT. The moderating influence of socio-demographic factors on the relationship between consumer psychographics and the attitude towards private label brands. J of Consumer Behav. 2013; 12: 423–435.

[38] Richardson PS, Jain AK, Dick A. Household store brand proneness: a framework. J of Retail. 1996; 72(2): 159–185.

[39] Cowart KO, Goldsmith RE. The influence of consumer decision-making styles on online apparel consumption by college students. Int J of Consum Stud. 2007; 31: 639–647.

[40] Lunn P, Lyons S. Behavioural economics and “vulnerable consumers”: a summary of evidence Dublin: Economic and Social Research Institute; 2010

[41] Lyon P. Editorial. Int J of Consum Stud. 2015; 39(4): 283.

[42] Nikou S. Mobile technology and forgotten consumers: The young-elderly. Int J of Consum Stud. 2015; 39(4): 294–304.

[43] Igbaria M, Parsuraman S. A path analytic study of individual characteristics, computer anxiety, and attitudes toward microcomputers. J of Manag. 1989; 15(3): 373–388.

[44] Bower GH, Hilgard ER. Theories of learning Englewood Cliffs, NJ: Prentice Hall; 1981.

[45] Norum PS. Student internet purchases. Fam and Consum Sci Res J. 2008; 4: 373–388.

[46] Lightner NJ. What users want in e-commerce design: effects of age, education and income. Ergonomics. 2003; 46: 153–168. doi: 10.1080/00140130303530 12554404 10.1080/00140130303530

[47] Seock YK, Bailey LR. The influence of college students' shopping orientations and gender differences on online information searches and purchase behaviours. Int J of Consum Stud. 2008 3 1;32(2):113–21.

[48] Hasan B. Exploring gender differences in online shopping attitude. Comput in Hum Behav. 2010 7 31; 26(4): 597–601.

[49] Garbarino E, Strahilevitz M. Gender differences in the perceived risk of buying online and the effects of receiving a site recommendation. J of Bus Res. 2004 7 31; 57(7): 768–75.

[50] Walther JB, D’Addario KP. The impacts of emoticons on message interpretation in computer-mediated communication. Soc Sci Comput Rev. 2001; 19(3): 324–347.

[51] Chevalier JA, Mayzlin D. The effect of word of mouth on sales: online book reviews. J of Mark Res. 2006; 43(3): 345–354.

[52] Duan W, Gu B, Whinston AB. The dynamics of online word-of-mouth and product sales: An empirical investigation of the movie industry. J of Retail. 2008; 84(2): 233–242.

[53] Gao J, Zhang C, Wang K, Ba S. Understanding online purchase decision making: The effects of unconscious thought, information quality, and information quantity. Decis Support Syst. 2012; 53(4): 772–781.

[54] Lix LM, Keselman JC, Keselman HJ. Consequences of assumption violations revisited: a quantitative review of alternatives to the one-way analysis of variance F test. Rev of Educational Res. 1996; 66: 579–619.

[55] Stevens JP. Applied multivariate statistics for the social sciences New York: Routledge; 2012.

