# Supplementary material for: Nudging to prevent the purchase of incompatible digital products online: An experimental study
Source: PLoS One. 2017 Mar 10;12(3):e0173333. doi: 10.1371/journal.pone.0173333 (PMC5345791; doi:10.1371/journal.pone.0173333)
Supplement: S1 Screenshots — (PDF) [file pone.0173333.s002.pdf]

-LINEEX- buy vip

Home | Products | Contact | Terms & Conditions

Remaining Credit: **27 ECUs** | Remaining Seconds: 571

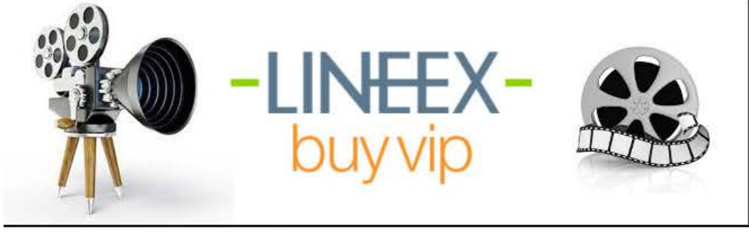

The best movies

Explore our available movies on streaming.

1 2

Categories

- Movies
- Games

Contact information

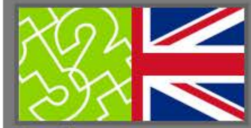

Lineex Shop
10 South Molton Street,
London, W1K 5QH
United Kingdom
+44 020 7499 8002
info@lineexshop.co.uk

Games

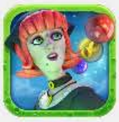

Bubble Witch

5.4 ECUs

Details

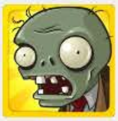

Plants versus Zombies

5.4 ECUs

Details

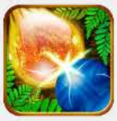

Zuma

5.4 ECUs

Details

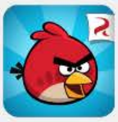

Angry Birds

5.4 ECUs

Details

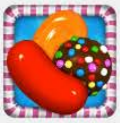

Candy crush

5.4 ECUs

Details

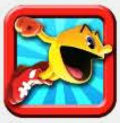

PAC-MAN DASH!

5.4 ECUs

Details

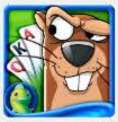

Fairway Solitarie

5.4 ECUs

Details

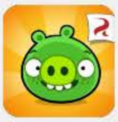

Bad piggies

5.4 ECUs

Details

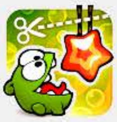

Cut the rope

5.4 ECUs

Details

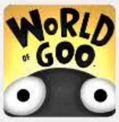

World of Goo

5.4 ECUs

Details

Movies

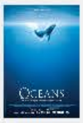

Oceans

5.4 ECUs

Details

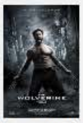

The Wolverine

5.4 ECUs

Details

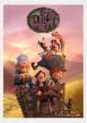

The Smoke Seller

5.4 ECUs

Details

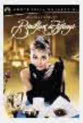

Breakfast at Tiffany's

5.4 ECUs

Details

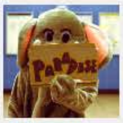

Coldplay - Paradise

5.4 ECUs

Details

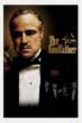

The Godfather

5.4 ECUs

Details

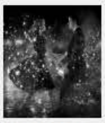

Nuit Blanche

5.4 ECUs

Details

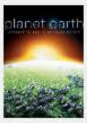

Planet Earth

5.4 ECUs

Details

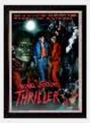

Thriller

5.4 ECUs

Details

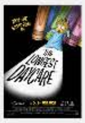

The Longest Daycare

5.4 ECUs

Details

Copyright © Lineex Shop | Designed by Lineex

-LINEEX- buyvip

Home | Products | Contact | Terms & Conditions

Remaining Credit: **27 ECUs** | Remaining Seconds: 230

Categories

- Movies
- Games

Contact information

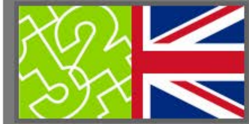

Lineex Shop
10 South Molton Street,
London, W1K 5QH
United Kingdom
+44 020 7499 8002
info@lineexshop.co.uk

Games

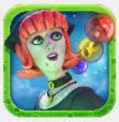

Bubble Witch

5.4 ECUs

Details

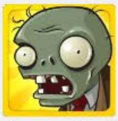

Plants versus Zombies

5.4 ECUs

Details

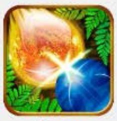

Zuma

5.4 ECUs

Details

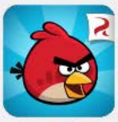

Angry Birds

5.4 ECUs

Details

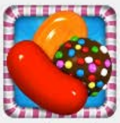

Candy crush

5.4 ECUs

Details

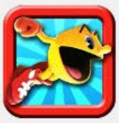

PAC-MAN DASH!

5.4 ECUs

Details

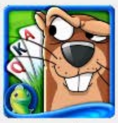

Fairway Solitarie

5.4 ECUs

Details

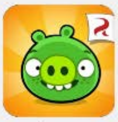

Bad piggies

5.4 ECUs

Details

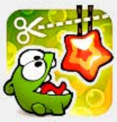

Cut the rope

5.4 ECUs

Details

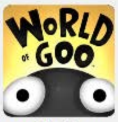

World of Goo

5.4 ECUs

Details

Copyright © Lineex Shop | Designed by Lineex

-LINEEX- buyvip

Categories

Movies
Games

Contact information

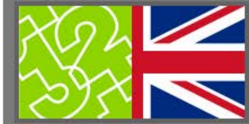

Lineex Shop
10 South Molton Street,
London, W1K 5QH
United Kingdom
+44 020 7499 8002
info@lineexshop.co.uk

Movies

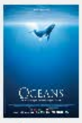

Oceans

5.4 ECUs

Details

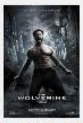

The Wolverine

5.4 ECUs

Details

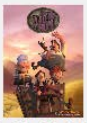

The Smoke Seller

5.4 ECUs

Details

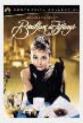

Breakfast at Tiffany's

5.4 ECUs

Details

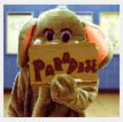

Coldplay - Paradise

5.4 ECUs

Details

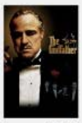

The Godfather

5.4 ECUs

Details

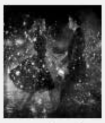

Nuit Blanche

5.4 ECUs

Details

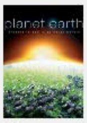

Planet Earth

5.4 ECUs

Details

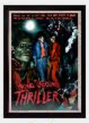

Thriller

5.4 ECUs

Details

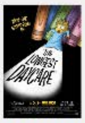

The Longest Daycare

5.4 ECUs

Details

Copyright © Lineex Shop | Designed by Lineex

-LINEEX- buyvip

Home | Products | Contact | Terms & Conditions

Remaining Credit: **27 ECUs** | Remaining Seconds: 260

Categories

- Movies
- Games

Contact information

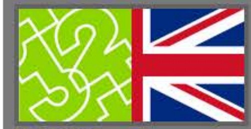

Lineex Shop
10 South Molton Street,
London, W1K 5QH
United Kingdom
+44 020 7499 8002
info@lineexshop.co.uk

Games

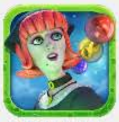

Bubble Witch

5.4 ECUs

Details

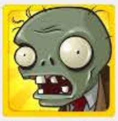

Plants versus Zombies

5.4 ECUs

Details

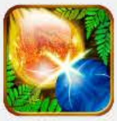

Zuma

5.4 ECUs

Details

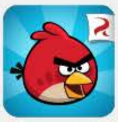

Angry Birds

5.4 ECUs

Details

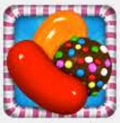

Candy crush

5.4 ECUs

Details

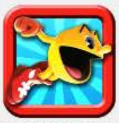

PAC-MAN DASH!

5.4 ECUs

Details

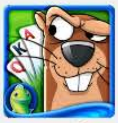

Fairway Solitarie

5.4 ECUs

Details

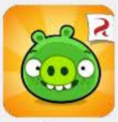

Bad piggies

5.4 ECUs

Details

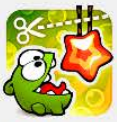

Cut the rope

5.4 ECUs

Details

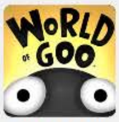

World of Goo

5.4 ECUs

Details

Movies

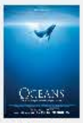

Oceans

5.4 ECUs

Details

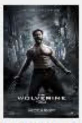

The Wolverine

5.4 ECUs

Details

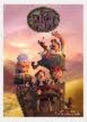

The Smoke Seller

5.4 ECUs

Details

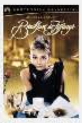

Breakfast at Tiffany's

5.4 ECUs

Details

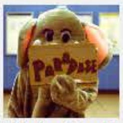

Coldplay - Paradise

5.4 ECUs

Details

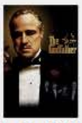

The Godfather

5.4 ECUs

Details

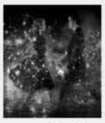

Nuit Blanche

5.4 ECUs

Details

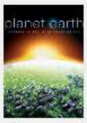

Planet Earth

5.4 ECUs

Details

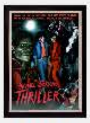

Thriller

5.4 ECUs

Details

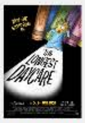

The Longest Daycare

5.4 ECUs

Details

Copyright © Lineex Shop | Designed by Lineex

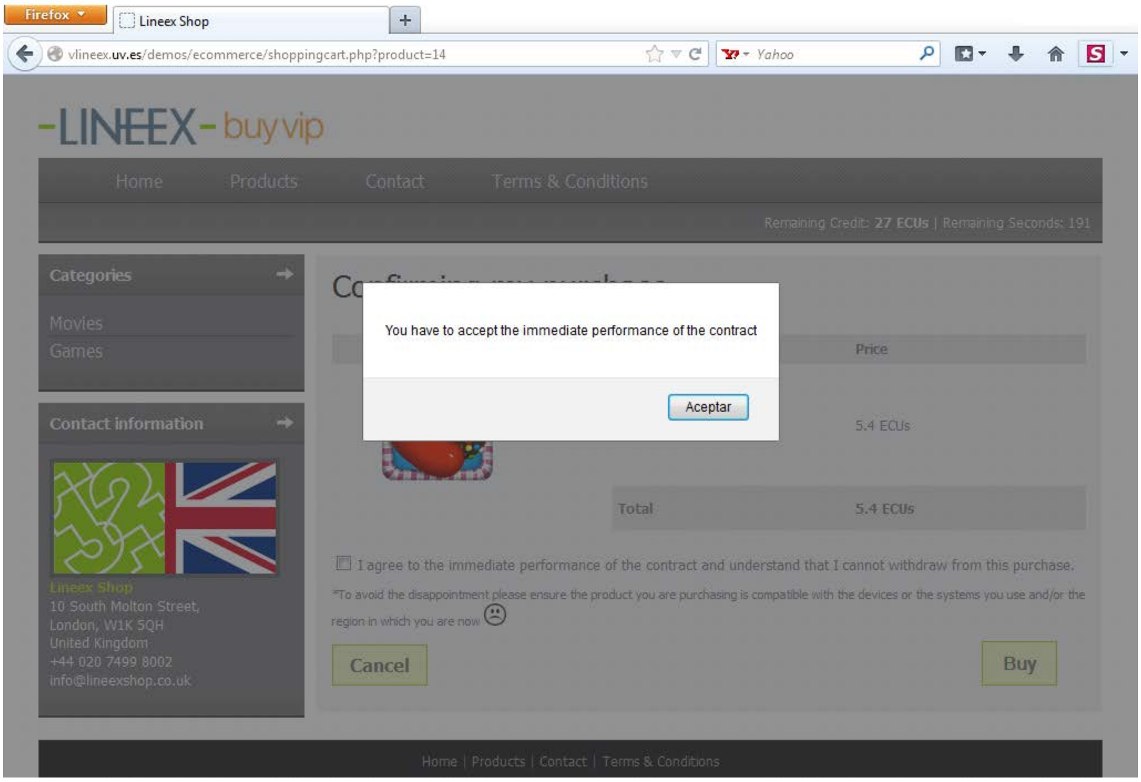
Firefox
Lineex Shop
vlineex.uv.es/demos/ecommerce/shoppingcart.php?product=14
Yahoo
-LINEEX- buy vip
Home
Products
Contact
Terms & Conditions
Remaining Credit: 27 ECUs | Remaining Seconds: 191
Categories
Movies
Games
Contact information
Lineex Shop
10 South Molton Street,
London, W1K 5QH
United Kingdom
+44 020 7499 8002
info@lineexshop.co.uk
You have to accept the immediate performance of the contract
Aceptar
Price
5.4 ECUs
Total
5.4 ECUs
I agree to the immediate performance of the contract and understand that I cannot withdraw from this purchase.
*To avoid the disappointment please ensure the product you are purchasing is compatible with the devices or the systems you use and/or the region in which you are now
Cancel
Buy
Home | Products | Contact | Terms & Conditions

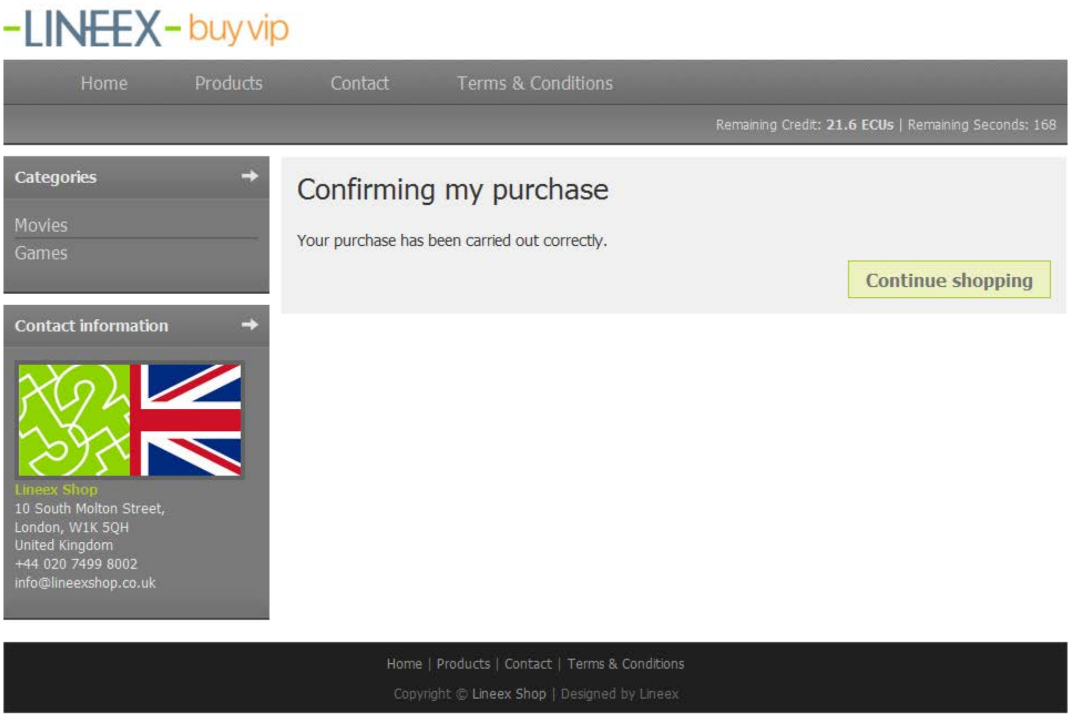
-LINEEX- buy vip
Home
Products
Contact
Terms & Conditions
Remaining Credit: 21.6 ECUs | Remaining Seconds: 168
Categories
Movies
Games
Contact information
Lineex Shop
10 South Molton Street,
London, W1K 5QH
United Kingdom
+44 020 7499 8002
info@lineexshop.co.uk
Confirming my purchase
Your purchase has been carried out correctly.
Continue shopping
Home | Products | Contact | Terms & Conditions
Copyright © Lineex Shop | Designed by Lineex

-LINEEX- buyvip

Home Products Contact Terms & Conditions

Remaining Credit: **27 ECUs** | Remaining Seconds: 214

Categories

Movies
Games

Contact information

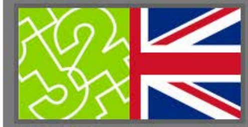

Lineex Shop
10 South Molton Street,
London, W1K 5QH
United Kingdom
+44 020 7499 8002
info@lineexshop.co.uk

Contact information

Postal address

Lineex Shop

10 South Molton Street,
London, W1K 5QH
United Kingdom
+44 020 7499 8002
info@lineexshop.co.uk

Phone +44 020 7499 8002
Email: info@lineexshop.co.uk

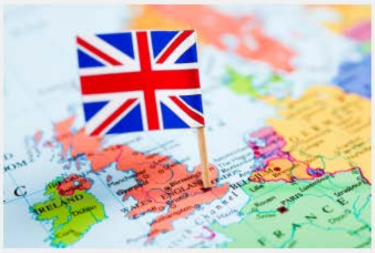

Home | Products | Contact | Terms & Conditions

Copyright © Lineex Shop | Designed by Lineex

Contact information

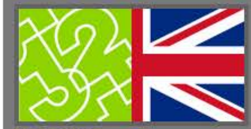

Lineex Shop
10 South Molton Street,
London, W1K 5QH
United Kingdom
+44 020 7499 8002
info@lineexshop.co.uk

Candy crush

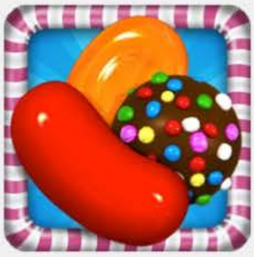

Price	5.4 ECUs
Genre	Arcade

Description

Welcome to the Candy Shop! Which one is your favorite? Crush candy to create special sweets such as striped candy, wrapped candy and rainbow candy. Combine these specials to create spectacular effects such as Super Stripe and Mega-Candy. The game can only be run on Windows. To play the game properly it is recommended to have a computer with some a minimum hardware requirements, such as 1 GB RAM, 256 MB graphics card, a 2.4 GHz processor and 500 MB or hard drive space.

Preview

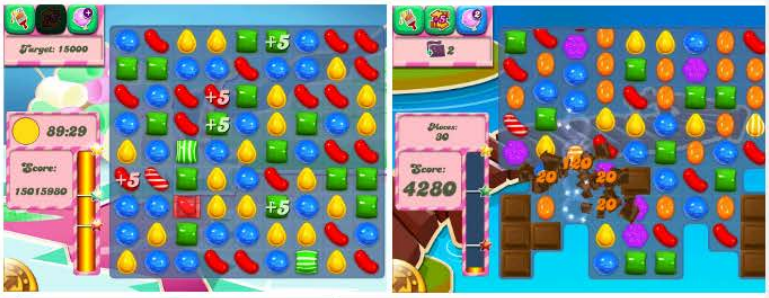

Compatible	Language Spanish	Recommended Hardware	2.4GHz Processor<br>500MB HD space

Back | Go to Buy

Reviews

"Quite frankly, we didn't expect to fall in love with this game. Excellent stuff."

Modojo , 2012-11-18

"Candy Crush Saga is as addictive as it is frustrating. The game starts off easy, but soon gets difficult and very challenging."

XGN , 2013-03-12

Users' comments

I started playing because a friend invited me to play. I started to play, and didn?t realize how much time actually passed before I ran out of lives. I can?t wait to play again.

Rjreyes34 , 2012-11-10

I can't stop playing this game. It's so addictive and I just want to get through all the puzzles. However minus 2 points because the amount of money they charge for things is ridiculously expensive. I guess I just have to wait for friends to help me out.... Collapse.

Wincew , 2013-05-13

Copyright © Lineex Shop | Designed by Lineex

Contact information

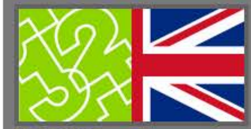

Lineex Shop
10 South Molton Street,
London, W1K 5QH
United Kingdom
+44 020 7499 8002
info@lineexshop.co.uk

Candy crush

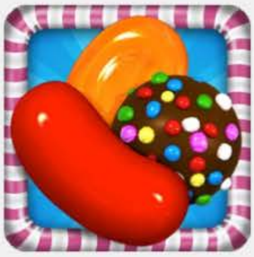

Price 5.4 ECUs

Genre Arcade

Description

Welcome to the Candy Shop! Which one is your favorite? Crush candy to create special sweets such as striped candy, wrapped candy and rainbow candy. Combine these specials to create spectacular effects such as Super Stripe and Mega-Candy. The game can only be run on Windows. To play the game properly it is recommended to have a computer with some a minimum hardware requirements, such as 1 GB RAM, 256 MB graphics card, a 2.4 GHz processor and 500 MB or hard drive space.

Preview

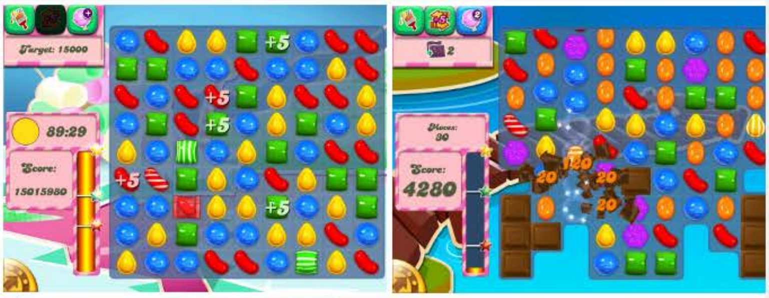

Back

Go to Buy

Reviews

"Quite frankly, we didn't expect to fall in love with this game. Excellent stuff."

Modojo , 2012-11-18

"Candy Crush Saga is as addictive as it is frustrating. The game starts off easy, but soon gets difficult and very challenging."

XGN , 2013-03-12

Users' comments

I started playing because a friend invited me to play. I started to play, and didn?t realize how much time actually passed before I ran out of lives. I can?t wait to play again.

Rjreyes34 , 2012-11-10

I can't stop playing this game. It's so addictive and I just want to get through all the puzzles. However minus 2 points because the amount of money they charge for things is ridiculously expensive. I guess I just have to wait for friends to help me out.... Collapse.

Wincew , 2013-05-13

Copyright © Lineex Shop | Designed by Lineex

-LINEEX- buyvip

Home | Products | Contact | Terms & Conditions

Remaining Credit: **27 ECUs** | Remaining Seconds: 441

Categories

Movies
Games

Contact information

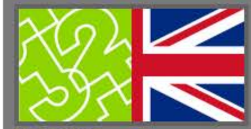

Lineex Shop
10 South Molton Street,
London, W1K 5QH
United Kingdom
+44 020 7499 8002
info@lineexshop.co.uk

Candy crush

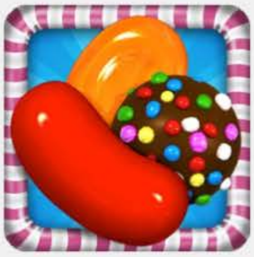

Price 5.4 ECUs

Genre Arcade

Description

Welcome to the Candy Shop! Which one is your favorite? Crush candy to create special sweets such as striped candy, wrapped candy and rainbow candy. Combine these specials to create spectacular effects such as Super Stripe and Mega-Candy. The game can only be run on Windows. To play the game properly it is recommended to have a computer with some a minimum hardware requirements, such as 1 GB RAM, 256 MB graphics card, a 2.4 GHz processor and 500 MB or hard drive space.

Preview

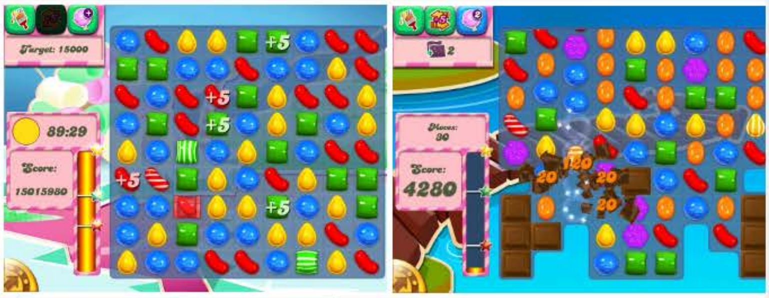

**Compatible**	Windows	**Language**	Spanish	**Recommended Hardware**	2.4GHz Processor<br>500MB HD space

Back | Go to Buy

Reviews

"Quite frankly, we didn't expect to fall in love with this game. Excellent stuff."

Modojo , 2012-11-18

"Candy Crush Saga is as addictive as it is frustrating. The game starts off easy, but soon gets difficult and very challenging."

XGN , 2013-03-12

Users' comments

I started playing because a friend invited me to play. I started to play, and didn?t realize how much time actually passed before I ran out of lives. I can?t wait to play again.

Rjreyes34 , 2012-11-10

I can't stop playing this game. It's so addictive and I just want to get through all the puzzles. However minus 2 points because the amount of money they charge for things is ridiculously expensive. I guess I just have to wait for friends to help me out.... Collapse.

Wincew , 2013-05-13

Copyright © Lineex Shop | Designed by Lineex

-LINEEX- buy vip

Remaining Credit: **18 ECUs** | Remaining Seconds: 150

Categories

- Movies
- Games

Contact information

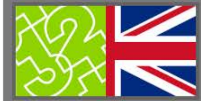

Lineex Shop
10 South Molton Street,
London, W1K 5QH
United Kingdom
+44 020 7499 8002
info@lineexshop.co.uk

The Wolverine

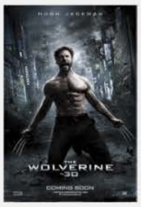

Price 3.6 ECUs

Genre Fantasy. Sci-Fi. Action | Superheroes. Based on Comic. Marvel Comics. Spin-off. Sequel

Description

Summoned to Japan by an old acquaintance, Wolverine becomes embroiled in a conflict that forces him to confront his own demons. The movie was directed by James Mangold in 2013 and produced by Marvel Enterprises / Twentieth Century Fox Film Corporation (USA). The movie can only be watched in Europe and has a length of 148 seconds. To watch the movie properly it is recommended to have a computer with some a minimum hardware requirements, such as 1 GB RAM, 256 MB graphics card, a 2.4 GHz processor and 500 MB or hard drive space.

Preview

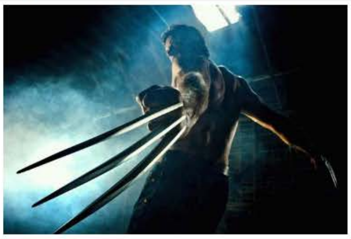

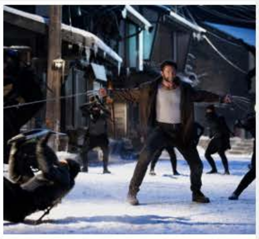

Compatible	Language Spanish	Length 148 seconds

Back | Go to Buy

Reviews

"In the glut of superhero movies hitting theaters, The Wolverine stands out for several reasons."

Scott Nash. Three Movie Buffs, *2013-07-30*

Users' comments

Just as comic-book movie fatigue was starting to set in, along comes The Wolverine to revive a moribund summer of superheroes.

Rene Rodriguez, *2013-07-26*

This film doesn't offer much in the way of excitement or suspense. Aside from a slightly unexpected plot development in the final minutes, it does things by the book.

Matthew Toomey, *2013-07-29*

Copyright © Lineex Shop | Designed by Lineex

-LINEEX- buy vip

Home Products Contact Terms & Conditions

Remaining Credit: **18 ECUs** | Remaining Seconds:

Categories

Movies
Games

Contact information

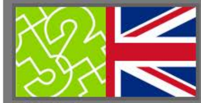

Lineex Shop
10 South Molton Street,
London, W1K 5QH
United Kingdom
+44 020 7499 8002
info@lineexshop.co.uk

The Wolverine

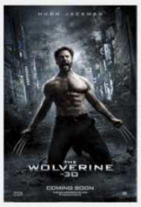

Price 3.6 ECUs

Genre Fantasy. Sci-Fi. Action | Superheroes. Based on Comic. Marvel Comics. Spin-off. Sequel

Description

Summoned to Japan by an old acquaintance, Wolverine becomes embroiled in a conflict that forces him to confront his own demons. The movie was directed by James Mangold in 2013 and produced by Marvel Enterprises / Twentieth Century Fox Film Corporation (USA). The movie can only be watched in Europe and has a length of 148 seconds. To watch the movie properly it is recommended to have a computer with some a minimum hardware requirements, such as 1 GB RAM, 256 MB graphics card, a 2.4 GHz processor and 500 MB or hard drive space.

Preview

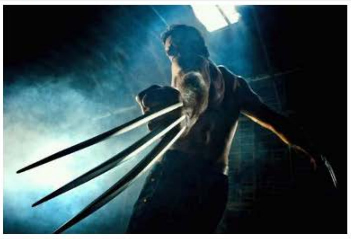

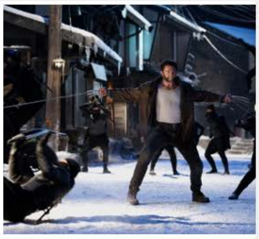

Back

Go to Buy

Reviews

"In the glut of superhero movies hitting theaters, The Wolverine stands out for several reasons."

Scott Nash. Three Movie Buffs, 2013-07-30

Users' comments

Just as comic-book movie fatigue was starting to set in, along comes The Wolverine to revive a moribund summer of superheroes.

Rene Rodriguez , 2013-07-26

This film doesn't offer much in the way of excitement or suspense. Aside from a slightly unexpected plot development in the final minutes, it does things by the book.

Matthew Toomey , 2013-07-29

Copyright © Lineex Shop | Designed by Lineex

-LINEEX- buy vip

Home Products Contact Terms & Conditions

Remaining Credit: **18 ECUs** | Remaining Seconds:

Categories

Movies
Games

Contact information

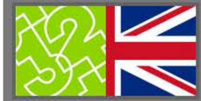

Lineex Shop
10 South Molton Street,
London, W1K 5QH
United Kingdom
+44 020 7499 8002
info@lineexshop.co.uk

The Wolverine

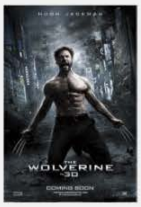

Price 3.6 ECUs

Genre Fantasy. Sci-Fi. Action | Superheroes. Based on Comic. Marvel Comics. Spin-off. Sequel

Description

Summoned to Japan by an old acquaintance, Wolverine becomes embroiled in a conflict that forces him to confront his own demons. The movie was directed by James Mangold in 2013 and produced by Marvel Enterprises / Twentieth Century Fox Film Corporation (USA). The movie can only be watched in Europe and has a length of 148 seconds. To watch the movie properly it is recommended to have a computer with some a minimum hardware requirements, such as 1 GB RAM, 256 MB graphics card, a 2.4 GHz processor and 500 MB or hard drive space.

Preview

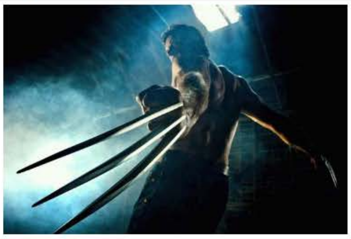

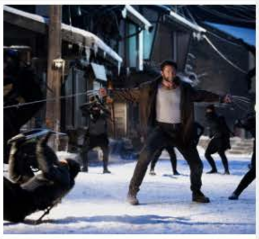

**Compatible**	Europe	**Language**	Spanish	**Length**	148 seconds

Back Go to Buy

Reviews

"In the glut of superhero movies hitting theaters, The Wolverine stands out for several reasons."

Scott Nash. Three Movie Buffs, *2013-07-30*

Users' comments

Just as comic-book movie fatigue was starting to set in, along comes The Wolverine to revive a moribund summer of superheroes.

Rene Rodriguez *, 2013-07-26*

This film doesn't offer much in the way of excitement or suspense. Aside from a slightly unexpected plot development in the final minutes, it does things by the book.

Matthew Toomey *, 2013-07-29*

Copyright © Lineex Shop | Designed by Lineex
